# Macronutrient and Bioactive Profiles of Donor Milk Differ in Commercial vs Non-Profit Milk Banks

**DOI:** 10.21203/rs.3.rs-7652186/v1

**Published:** 2025-10-07

**Authors:** Bridget Young, Tanner Tarkleson, Laura Cole, Manisha Srivastava, Lori Scholer, Carrie Gale, Jeffrey Meyers, Carl D’Angio, Danielle Ackley

**Affiliations:** University of Rochester School of Medicine and Dentistry; University of Rochester School of Medicine and Dentistry; University of Rochester School of Medicine and Dentistry; University of Rochester School of Medicine and Dentistry; University of Rochester School of Medicine and Dentistry; University of Rochester Medical Center; University of Rochester Medical Center; University of Rochester

## Abstract

**Objective:**

The objective was to explore variation of macronutrient and bioactive content between levels of human milk-derived human milk fortifier (HM-HMF), and between donor human milk (DHM) from commercial vs non-profit banks.

**Study Design:**

We analyzed the concentration of macronutrients, Immunoglobulin A (IgA), and cortisol in multiple lots of each HM-HMF product (20, 24, 26, 28, and 30 kcal/oz) using multiple methods.

**Result:**

At each level of caloric density (p<0.0001), protein, carbohydrate, and caloric concentration in HM-HMF increased with minor exceptions while fat and cortisol concentrations did not differ. Total IgA concentrations differed by product type (p <0.0001). Protein concentration did not differ between commercial and non-profit donor milk banks while carbohydrates, fat, calories, and cortisol were higher, and IgA was lower. Lot-to-lot variability of all DHM components was lower in commercial DHM.

**Conclusion:**

This study expands data on the variability in the composition of DHM originating from various sources.

## INTRODUCTION

The American Academy of Pediatrics recommends pasteurized donor human milk (DHM) as the preferred option to feed a premature infant when mother’s own milk is unavailable (1). The Human Milk Banking Association of North America (HMBANA) is a non-profit organization of several donor milk banks across North America that provides DHM primarily to premature infants in Neonatal Intensive Care Units (NICUs) due to its protective effects against necrotizing enterocolitis (2, 3). DHM may necessitate additional fortification, as the average caloric and protein content of DHM from non-profit milk banks has been reported as 18 kcal/oz and 0.74 g/dL, respectively (4, 5) in comparison to the assumed composition of premature mother’s milk at 20 kcal/oz and of 1.4 g/dL protein.

There are currently no studies analyzing the macronutrient content or characterizing bioactive components of DHM from commercial donor milk banks, although many such banks provide a nutrient label on the DHM produced. It can, however, be hypothesized that there is variability in macronutrients between product lots given that the FDA allows for calorie content to be between 100–105% and protein content to be within 95–105% of the labeled value (6). The variability in labeled macronutrients and unlabeled bioactive components of DHM (and DHM-derived products) from commercial vs non-profit sources has never been reported. To fill this gap, we investigated the mean and variability of the macronutrient and bioactive (cortisol, and immunoglobulin A (IgA)) content of DHM and human milk-derived products from a commercial company compared to DHM from a non-profit milk bank. Our overall goal is to provide detailed information to NICU providers to support optimizing feeding of premature infants.

## METHODS

### Product Acquisition and Preparation:

Lots of DHM, and DHM-derived concentrated products (both human milk-derived human milk fortifiers and ready-to-feed product) were obtained from the manufacturer. For products with a caloric density of 24, 26, and 28 kcal/oz, both fortifier and ready-to-feed product lots were obtained (the 30 kcal/oz product is available only as +10 kcal/oz fortifier). All human milk fortifier obtained was reconstituted in the same lot of 20 kcal/oz DHM, as per the manufacturer’s instructions.

Skim milk was prepared by centrifuging samples at 10,000g for 10 minutes at 4°C. Skim milk was extracted without disturbing the separated fat or any precipitate. All samples were stored at −80°C until analyses.

### Sample Analysis:

Macronutrients (total fat, protein, and carbohydrate), as well as calculated caloric density were assessed via mid-infrared spectroscopy (Miris AB, Uppsala Sweden). Samples were run in duplicate. True protein is reported in this study.

Total protein was also measured in skim milk via the Bradford Assay (BioRad: 50000001, Hercules, CA). Skim milk from DHM was diluted 1:1,000 in distilled water. Skim milk from concentrated human milk product (>20 kcal/oz) was diluted 1:1,500 in distilled water. Diluted samples were analyzed according to the manufacturer’s instructions.

Measurement of macronutrients was also conducted by an independent third-party food analysis lab (Eurofins, Madison, WI). This lab measured total nitrogen using a combustion-detection technique to estimate total protein. Total fat was measured using a base hydrolysis technique. Total ash was determined by gravimetric assessment of the ash remaining after organic matter was ignited at 550°C in an electric furnace. Total sample moisture was determined after vacuum drying at 100°C for 5–5.5 hours. Total carbohydrate was calculated using a formula accounting for measurement of protein, fat, ash, and moisture (Official Methods of Analysis of AOAC INTERNATIONAL, 18th Ed., Methods 925.09 and 926.08, AOAC INTERNATIONAL, Gaithersburg, MD, USA,(2005). (Modified). Total caloric content was calculated from carbohydrate, protein, and fat measurements, assuming carbohydrate and protein = 4 kcal/g and fat = 9 kcal/g.

Lactose and galactose were measured using a calorimetric enzymatic assay (Sigma Aldrich: MAK017, St Louis MO). For assessment of galactose, skim milk samples were diluted 1:10, and then analyzed according to the manufacturer’s instructions for measuring galactose. For assessment of lactose, skim milk samples were diluted 1:2,000, and then analyzed according to the manufacturer’s instructions for measuring lactose.

Glucose was measured in skim milk via a peroxidase assay (Sigma Aldrich: GAGO20, St Louis MO). Skim milk samples were diluted 1:2 in water and then analyzed according to the manufacturer’s instructions.

Total IgA measured in skim milk via custom ELISA, as described in previous studies (7, 8).

Cortisol was measured in whole milk samples using a method previously validated in human milk (9) that utilizes an enzyme-linked immunosorbent assay marketed for the detection of salivary cortisol (DRG International, Inc., Springfield, NJ, SKU: SLV2930R) and incorporates sonication of whole milk samples.

### Determination of comparison values of non-profit donor human milk from previously published studies

To identify relevant comparison values from non-profit DHM samples, a comprehensive literature search was conducted using PubMed. The search included peer-reviewed articles published between 2015 and 2025. Keywords for search included “human milk bank”, “macronutrients”, “cortisol”, and “IgA” in combination with relevant Boolean operators.

Included studies provided quantitative values for components of human milk from a non-profit donor milk bank using the same analytical techniques we used, studies with insufficient data or from non-peer reviewed sources were excluded. Values were extracted directly from tables, figures, or text and preference was given to studies with larger sample sizes.

Published data of a non-profit DHM that used the same methodology as we used for analysis of samples from a commercial DHM were available for all analytes, with the exception of cortisol. We assessed cortisol in human milk samples using an ELISA. Two other studies measuring cortisol in DHM from non-profit milk banks utilized a different immune-based platform (Luminex, Millipore Sigma, St Louis, MO) (10) and liquid chromatography mass spectrometry (11).

### Statistical Analyses

Unless otherwise noted, results are presented as mean ± standard deviation (SD). Descriptive statistics included mean, standard deviation, minimum, and maximum. Comparisons between different methods assessing the same analyte were conducted using Bland-Altman plots and statistics. Differences in concentrations between product type (level of kcal/oz) were analyzed via ANOVA with pairwise comparisons between individual groups performed using student t-tests. Average analyte comparisons between commercial and non-profit DHM were compared via t-test using measured means and standard deviations for commercial DHM and published means and standard deviations and sample sizes for non-profit DHM. Similarly, differences in variance between the standard deviations of measured results from commercial DHM and published standard deviations of analyses of non-profit DHM were compared via F-test.

## RESULTS

Table 1 summarizes the studies identified for analogous measurement of macronutrients, cortisol, and IgA in DHM from a non-profit milk bank.

### Protein Composition of Human Milk Products:

Table 2 provides the average protein concentration measured in the DHM products obtained.

**Figure 1.**
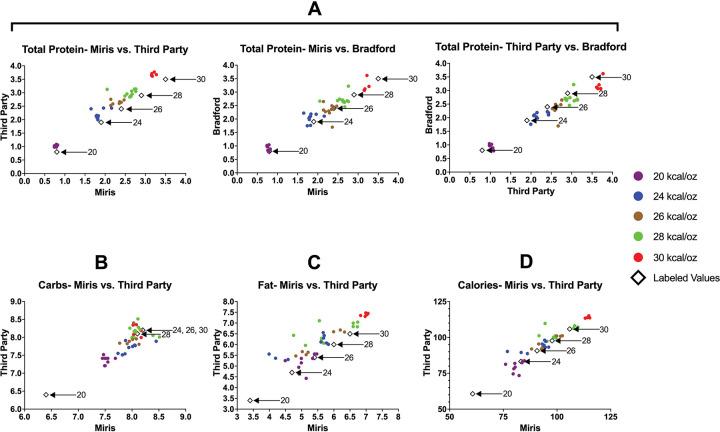
Comparison of macronutrient values from various measurement techniques to each other and labeled value. The macronutrient values of all product types (20, 24, 26, 28, and 30 kcal/oz, each in different colors) are plotted according to measurement technique. Each dot represents an individual product lot. A) Comparison of protein (g/100mL) as measured by Miris (mid-infrared spectroscopy, true protein reported), the Bradford assay, and a third-party assay based on total nitrogen. Comparison of total carbohydrate (g/100mL) (B), fat (g/100mL) (C), and caloric content (per 100 mL) (D) as measured by Miris (mid-infrared spectroscopy vs. third-party food analysis lab).

Protein concentration as measured by mid-infrared spectroscopy significantly increased with each level of caloric density (p<0.0001). Protein concentration detected via assessment of total nitrogen (via third party lab) was, on average, 0.387 g/dL higher than concentration detected via mid-infrared spectroscopy (p < 0.0001). Protein concentration detected via the Bradford assay was, on average, 0.131 g/dL higher than concentration detected via mid-infrared spectroscopy (p = 0.0027). Figure 1A presents the protein concentrations as assessed via different methodologies alongside the products’ labeled protein concentrations.

A study (5) of 123 DHM lots obtained from a non-profit donor milk bank assessed protein via mid-infrared spectroscopy and reported the average true protein concentrations to be 0.74 ± 0.14 g/dL. This average did not differ from the mean true protein of the DHM obtained from a commercial milk bank. The standard deviation of the protein concentrations from the commercial DHM was significantly lower than that of the non-profit DHM (p < 0.001, Table 1).

### Carbohydrate Composition of Human Milk Products:

Carbohydrate (via mid-infrared spectroscopy) concentration significantly increased with each level of caloric density (p<0.0001) aside from the 30 kcal/oz product, which was similar to the 28 kcal/oz product (Table 3). Carbohydrate concentration detected by a third-party lab was not significantly different than that measured via mid-infrared spectroscopy. Figure 1B presents the carbohydrate concentrations alongside the products’ labeled carbohydrate concentration.

A study (5) of 123 DHM lots obtained from a non-profit donor milk bank assessed carbohydrate concentration via mid-infrared spectroscopy and reported the average total carbohydrate concentrations to be 7.0 ± 0.2 g/dL. Compared to this value, the mean total carbohydrate of DHM obtained from a commercial milk bank was significantly higher (p<0.0001). The standard deviation of the carbohydrate concentrations from the commercial DHM was significantly lower than that of the non-profit DHM (p = 0.001, Table 1).

Similar to the total carbohydrate measurements, lactose and galactose concentrations increased as caloric density increased. Glucose concentrations were similar across product types. There have not been previous reports of lactose, glucose, or galactose measurements of DHM from non-profit milk banks using similar methods.

### Fat and Caloric Composition of Human Milk Products:

Fat (via mid-infrared spectroscopy) concentration did not significantly increase with each level of caloric density (Table 2). Fat concentration detected by a third-party lab was, on average, 0.49 g/100mL higher than concentration detected via mid-infrared spectroscopy (p < 0.001). Figure 1C presents the fat concentrations alongside the products’ labeled fat concentration.

A study (5) of 123 DHM lots obtained from a non-profit donor milk bank assessed fat concentration via mid-infrared spectroscopy and reported the average fat concentrations to be 2.94 ± 0.63 g/dL. Compared to this value, the mean total fat of the DHM obtained from a commercial milk bank (also assessed via mid-infrared spectroscopy) was significantly higher (p<0.0001). The standard deviation of the fat concentrations from the commercial DHM was significantly lower than that of the non-profit DHM (p = 0.015, Table 1).

Measured caloric concentration (via mid-infrared spectroscopy) was on average 10.4 kcal/100mL higher than the labeled value. Measured caloric concentration generally increased with caloric density (p<0.0001), aside from the 28 kcal/oz product which did not differ from the 26 kcal/oz product (Table 2). Caloric concentration detected by a third-party lab was, on average, 3.60 kcal/100mL higher than concentration detected via mid-infrared spectroscopy (p < 0.001). Figure 1D presents the caloric density of each product alongside the products’ labeled caloric value.

A study (5) of 123 DHM lots obtained from a non-profit donor milk bank assessed caloric concentration via mid-infrared spectroscopy and reported the average caloric concentration to be 17.6 ± 1.7 kcal/oz. Compared to this value, the mean caloric density of the DBM obtained from a commercial milk bank (also assessed via mid-infrared spectroscopy) was significantly higher (p<0.0001). The standard deviation of the caloric content from the commercial DHM was significantly lower than that of the non-profit DHM (p = 0.013, Table 1).

### Lactose/Fat Ratio

The lactose/fat ratio (as measured via mid-infrared spectroscopy) was significantly lower in the 30 kcal/oz product type compared to the 20, 24, and 26 kcal/oz products (p < 0.05), ranging from 1.48 ± 0.08 in the 24 kcal/oz product to 1.13 ± 0.11 in the 30 kcal/oz product.

### Cortisol

Table 1 presents the total cortisol concentrations detected in the different DHM-derived products. Measured cortisol concentrations did not differ across different product types. A study (10) of 52 DHM lots obtained from a non-profit donor milk bank assessed cortisol concentration using a different ELISA platform (Milliplex by Luminex, Millipore Sigma, St Louis, MO) and reported the average cortisol concentration to be 2.84 ± 3.5 ng/mL. Compared to this value, the mean total cortisol of the DHM from the commercial milk bank was significantly higher (p = 0.0004). The standard deviation of the cortisol concentrations from the commercial DHM bank was significantly lower than that of the non-profit donor milk bank (p < 0.01).

### IgA

Table 1 presents the total IgA concentrations detected in the different DHM-derived products. Measured total IgA (via ELISA) concentrations were different by product type (p<0.0001), with the 30 and 28 kcal/oz products exhibiting higher IgA concentrations than the 20 and 26 kcal/oz products (p<0.02; Table 1). A study (7) of 126 DHM lots obtained from a non-profit donor milk bank assessed total IgA concentration (using the same ELISA) reported the average total IgA concentration to be 230 ± 100 μg/mL. Compared to this value, the mean total IgA of the DHM obtained from a commercial milk bank was significantly lower (p < 0.0001). The standard deviation of the total IgA concentrations from the commercial DHM was also significantly lower than that of the non-profit DHM (p < 0.001).

## DISCUSSION

This study demonstrates that the base (20 kcal/oz) DHM product from a commercial milk bank had significantly less variability across all categories as compared to DHM from a non-profit milk bank(s). Additionally, the amount of fat, calories, carbohydrates, and cortisol in DHM from a commercial milk bank was significantly greater than DHM from a non-profit milk bank(s). In products from a commercial milk bank, the concentration of carbohydrate, fat, and total calories tended to be higher than the labeled value.

Meeting premature infants’ protein needs is often a challenge to supporting optimal growth. Here we show that DHM from commercial and non-profit milk banks provide equivalent protein concentrations. However, there is less lot-to-lot variability in DHM obtained from a commercial milk bank. Human milk has a relatively high non-protein nitrogen content (20–25%, primarily from urea (12, 13)), and thus estimates based on total nitrogen over-estimate protein that is bioavailable to the infant. In contrast, cow’s milk has a lower non-protein nitrogen of 5% (12). Protein values placed on a food label (including those of human milk-derived products) are required to be based on total nitrogen content, which is known to be an overestimate of bioavailable protein in human milk. “True protein” form the Miris accounts for non-protein nitrogen by applying a set adjustment value to the measured crude protein value. The Bradford assay utilizes Coomassie Brilliant Blue G-250 dye, which binds certain amino acids, to estimate protein. This explains why the total-nitrogen estimates of protein content were higher than that of the Bradford assay. It is important to note that if any filtration is utilized to generate concentrated DHM-derived products, then these products will likely have lower urea content as caloric density increases, as urea will be lost during any filtration. Filtration will decrease the percentage of non-protein nitrogen, indicating that: as the caloric content of DHM-fortified product increases, the Miris “true protein” is likely to be less accurate and an under-estimate of true protein content. Protein fortification of human milk results in better growth outcomes (14), and higher protein intakes (between 3.5 to 4.0 g/kg/d) may promote optimal weight gain and developmental outcomes (15) in premature infants. As such, understanding the nuances of non-protein nitrogen and how to interpret labeled protein content compared to protein assessed via different methods can have critical implications on how milk is fortified for premature infants.

In this study, we found that fat content in DHM-derived products was consistently higher than the labeled value, regardless of methodology used. Fat concentrations were higher, with less variability in DHM from a commercial compared to non-profit milk bank. This resulted in higher caloric content in milk from a commercial milk bank as well. The caloric difference between DHM from this commercial vs non-profit donor milk bank was quite large at 6.4 kcal/oz. This large difference suggests it may be prudent for feeding protocols to take into account source of DHM used when making feeding prescriptions for premature infants.

The carbohydrate content of concentrated DHM products increased with the degree of fortification up to 28 kcal/oz. This measurement includes both digestible carbohydrates and human milk oligosaccharides (HMOs). It is noteworthy that the concentrations of lactose did not increase with the degree of fortification, which could potentially mean that the more concentrated DHM-product may have higher concentrations of HMO’s. This is worthy of future research.

This is the first study to compare concentrations of two bioactive compounds between DHM from a commercial vs a non-profit milk bank. IgA was chosen as it is the most common immunoglobulin in human milk, may protect against infant illness (16–18), and is connected to lower measures of infant illness in premature infants (8). Furthermore, it is a protein and is detected in the skim portion of milk. Human milk cortisol has been linked with infant weight gain and growth patterns (19, 20), development of infant temperament (21–23), and may contribute to the development of infant circadian rhythm (24). Cortisol, unlike IgA, is fat-soluble and detected in the fat component of milk. Thus, it is not surprising that milk from the commercial milk bank, which had elevated fat concentrations, had higher concentrations of cortisol. However, comparison of cortisol concentrations between these studies may not be prudent given different immunoassays were used in each study. In this study, we find that IgA concentrations were higher in DHM from a non-profit compared to a commercial milk bank. HMBANA utilizes Holder pasteurization compared to vat pasteurization. However, these methods are similar enough that this does not completely explain the difference in the final concentrations.

This study is the first to compare the average and variability of both macronutrients and bioactive components of DHM from a commercial vs non-profit milk bank(s). We include assessment of both fat-soluble (cortisol) and soluble bioactive (IgA) components of milk. We have a relatively large sample size of individual lots and incorporate larger data from studies of HMBANA DHM. An additional strength is the use of multiple methods to assess each of the macronutrients in milk. This allows for a depth of interpretation that accounts for shortcomings in individual methodologies, particularly the Miris, which has limitations in accuracy and resolution of carbohydrates and protein. A limitation is our lack of data regarding HMO concentrations in these samples. Additionally, this dataset precludes us from linking any variation in milk composition with infant outcomes.

## CONCLUSIONS

This study provides novel data on the variability in the composition of DHM originating from various sources. When mother’s milk is unavailable, DHM from any milk bank is a preferable source of nutrition for premature infants compared to infant formula. This work provides valuable insight for providers caring for very premature infants regarding the variable considerations necessary to ensure prescribed enteral nutritional values are met.

## Supplementary Material

Tables

Table 1 and 2 are available in the Supplementary Files section.

Supplementary Files

This is a list of supplementary files associated with this preprint. Click to download.

• Table1.pptx

• Table2.pptx

## Abbreviations

DHM: Donor Human Milk
HMBANA: Human Milk Banking Association of North America
HM-HMF: Human Milk-derived Human Milk Fortifiers
